# Supplementary material for: Gender Differences in the Risk for Incident Non-Alcoholic Fatty Liver Disease According to the Transition of Abdominal Obesity Status: A 16-Year Cohort Study
Source: Nutrients. 2023 Jun 25;15(13):2880. doi: 10.3390/nu15132880 (PMC10346349; doi:10.3390/nu15132880)

Supplementary materials

Table S1. Definition of abdominal obesity status over time

Baseline (2001-2002)	First follow-up (2003-2004)	Group	Label
Normal	AO	1	Persistent leanness
AO	Normal	2	Improved from abdominal obesity
Normal	AO	3	Progressed to abdominal obesity
AO	AO	4	Persistent abdominal obesity

Table S2. Pairwise comparison p-value from unadjusted model and fully adjusted model over two-year follow-up

		Unadjusted model					Fully adjusted model*				
		HR (95% CI)	(1)	(2)	(3)	(4)	HR (95% CI)	(1)	(2)	(3)	(4)
**Total**	Persistent leanness	ref	ref				ref	ref			
	Improved from abdominal obesity	1.39 (1.13-1.72)	0.002	ref			1.06 (0.84–1.33)	0.637	ref		
	Progressed to abdominal obesity	2.26 (1.97-2.61)	<.001	<.001	ref		1.73 (1.48–2.02)	<.001	<.001	ref	
	Persistent abdominal obesity	2.56 (2.26-2.89)	<.001	<.001	0.154	ref	1.33 (1.13–1.57)	<.001	0.072	0.006	ref
**Men**	Persistent leanness	ref	ref				ref	ref			
	Improved from abdominal obesity	1.91 (1.32-2.77)	0.001	ref			1.47 (0.99–2.18)	0.055	ref		
	Progressed to abdominal obesity	2.25 (1.77-2.86)	<.001	0.457	ref		1.60 (1.22–2.09)	<.001	0.716	ref	
	Persistent abdominal obesity	2.78 (2.14-3.61)	<.001	0.094	0.211	ref	1.21 (0.87–1.69)	0.253	0.419	0.142	ref
**Women**	Persistent leanness	ref	ref				ref	ref			
	Improved from abdominal obesity	1.33 (1.03-1.73)	0.029	ref			0.93 (0.71–1.24)	0.628	ref		
	Progressed to abdominal obesity	2.38 (2.00-2.84)	<.001	<.001	ref		1.78 (1.47–2.16)	<.001	<.001	ref	
	Persistent abdominal obesity	2.70 (2.33-3.12)	<.001	<.001	0.215	ref	1.36 (1.12–1.65)	0.002	0.015	0.017	ref

*Adjusted for age, sex (in total population), body mass index, smoking, physical activity, alcohol drinking, total energy intake, mean blood pressure, glycosylated hemoglobin, total cholesterol, high-sensitivity C-reactive protein, and alanine aminotransferase.

Table S3. Cox proportional hazards regression analysis for incident non–alcoholic fatty liver disease according to abdominal obesity patterns

		Unadjusted		Model 1		Model 2		Model 3	
		HR (95% CI)	p–value	HR (95% CI)	p–value	HR (95% CI)	p–value	HR (95% CI)	p–value
**Total**	Persistent lean WC	ref		ref		ref		ref	
	Improved abdominal obesity	1.48 (1.16–1.89)	0.002	1.14 (0.88–1.46)	0.323	1.16 (0.89–1.50)	0.278	1.15 (0.89–1.50)	0.292
	Progressed to abdominal obesity	2.32 (2.00–2.70)	<.001	1.80 (1.54–2.11)	<.001	1.78 (1.51–2.10)	<.001	1.77 (1.50–2.09)	<.001
	Persistent abdominal obesity	2.44 (2.09–2.84)	<.001	1.30 (1.10–1.62)	0.003	1.29 (1.05–1.59)	0.015	1.33 (1.08–1.63)	0.007
**Men**	Persistent lean WC	ref		ref		ref		ref	
	Improved abdominal obesity	2.06 (1.35–3.13)	0.001	1.37 (0.88–2.11)	0.161	1.33 (0.85–2.08)	0.217	1.33 (0.85–2.10)	0.213
	Progressed to abdominal obesity	1.96 (1.49–2.59)	<.001	1.46 (1.10–1.94)	0.009	1.48 (1.10–2.00)	0.011	1.50 (1.11–2.03)	0.009
	Persistent abdominal obesity	2.23 (1.59–3.14)	<.001	1.08 (0.73–1.59)	0.692	1.01 (0.67–1.52)	0.973	1.10 (0.72–1.66)	0.668
**Women**	Persistent lean WC	ref		ref		ref		ref	
	Improved abdominal obesity	1.45 (1.07–1.96)	0.017	1.07 (0.79–1.46)	0.652	1.07 (0.78–1.48)	0.675	1.06 (0.77–1.47)	0.716
	Progressed to abdominal obesity	2.72 (2.26–3.28)	<.001	1.99 (1.64–2.42)	<.001	1.94 (1.59–2.38)	<.001	1.92 (1.57–2.35)	<.001
	Persistent abdominal obesity	2.78 (2.33–3.36)	<.001	1.42 (1.13–1.78)	0.003	1.38 (1.08–1.77)	0.010	1.41 (1.10–1.81)	0.006

Model 1: adjusted for age, sex (in total population), and body mass index, Model 2: Model 1 plus smoking, physical activity, alcohol drinking, total energy intake. Model 3: Model 2 plus smoking, physical activity, alcohol drinking, total energy intake, mean blood pressure, glycosylated hemoglobin, total cholesterol, high–sensitivity C–reactive protein, and alanine aminotransferase.

Abbreviations: WC, waist circumference; HR, hazard ratio; CI, confidence interval.

Table S4. Pairwise comparison p–value from unadjusted model and fully adjusted model during first four years of follow–up

		Unadjusted model					Full adjusted model				
		HR (95% CI)	(1)	(2)	(3)	(4)	HR (95% CI)	(1)	(2)	(3)	(4)
**Total**	Persistent lean WC	ref	ref				ref	ref			
	Improved abdominal obesity	1.48(1.16–1.89)	0.002	ref			1.15 (0.89–1.50)	0.292	ref		
	Progressed to abdominal obesity	2.32(2.00–2.70)	<.001	0.001	ref		1.77 (1.50–2.09)	<.001	0.003	ref	
	Persistent abdominal obesity	2.44(2.09–2.84)	<.001	<.001	0.627	ref	1.33 (1.08–1.63)	0.007	0.344	0.008	ref
**Men**	Persistent lean WC	ref	ref				ref	ref			
	Improved abdominal obesity	2.06(1.35–3.13)	0.001	ref			1.33 (0.85–2.10)	0.213	ref		
	Progressed to abdominal obesity	1.96(1.49–2.59)	<.001	0.845	ref		1.50 (1.11–2.03)	0.009	0.656	ref	
	Persistent abdominal obesity	2.23(1.59–3.14)	<.001	0.759	0.541	ref	1.10 (0.72–1.66)	0.668	0.488	0.182	ref
**Women**	Persistent lean WC	ref	ref				ref	ref			
	Improved abdominal obesity	1.45(1.07–1.96)	0.017	ref			1.06 (0.77–1.47)	0.716	ref		
	Progressed to abdominal obesity	2.72(2.26–3.28)	<.001	0.001	ref		1.92 (1.57–2.35)	<.001	<.001	ref	
	Persistent abdominal obesity	2.80(2.33–3.36)	<.001	<.001	0.804	ref	1.41 (1.10–1.81)	0.006	0.117	0.014	ref

*Adjusted for age, sex (in total population), body mass index, smoking, physical activity, alcohol drinking, total energy intake, mean blood pressure, glycosylated hemoglobin, total cholesterol, high-sensitivity C-reactive protein, and alanine aminotransferase.

Abbreviations: WC, waist circumference; HR, hazard ratio; CI, confidence interval.

Figure S1. Cumulative incidence of NAFLD according to abdominal obesity patterns by setting the accrual time from baseline to second follow-up time. (A)Total, (B) Men, and (C) Women.

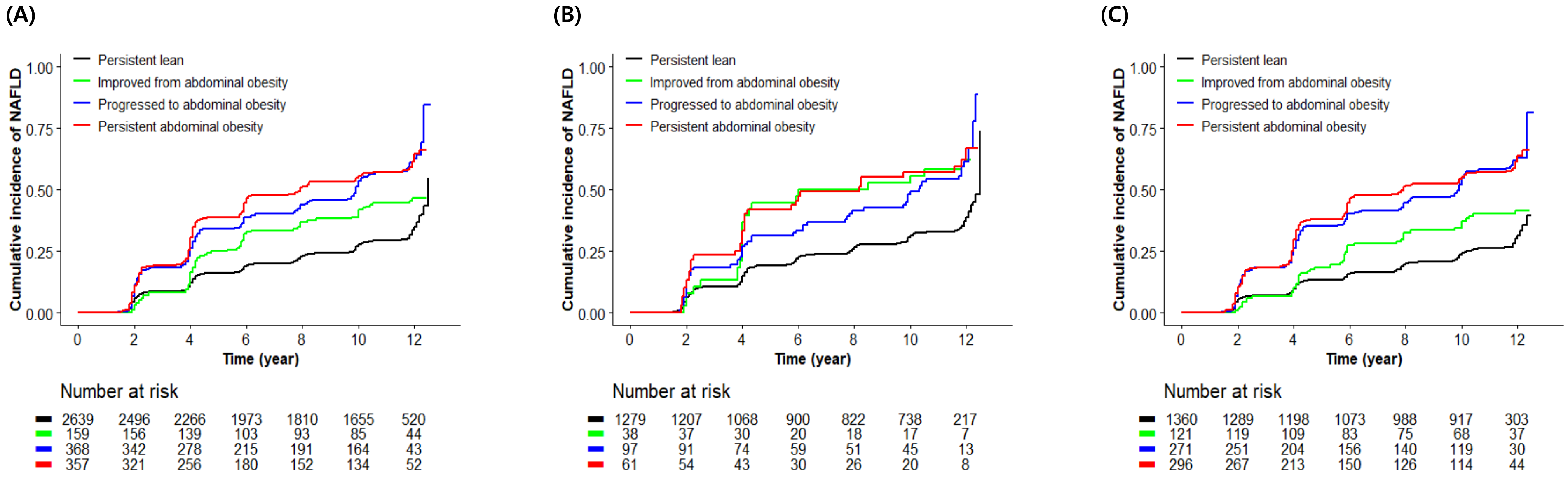

Supplement: Supplementary file 1 [file nutrients-15-02880-s001.zip › nutrients-2447515-supplementary.pdf]
